# The effects of L-carnitine supplementation on inflammation, oxidative stress, and clinical outcomes in critically Ill patients with sepsis: a randomized, double-blind, controlled trial

**DOI:** 10.1186/s12937-024-00934-4

**Published:** 2024-03-06

**Authors:** Mahdi Keshani, Babak Alikiaii, Zahra Babaei, Gholamreza Askari, Zahra Heidari, Manoj Sharma, Mohammad Bagherniya

**Affiliations:** 1 Nutrition and Food Security Research Center and Department of Community Nutrition, School of Nutrition and Food Science, Isfahan University of Medical Sciences, Isfahan, Iran; 2 Anesthesia and Critical Care Research Center, Isfahan University of Medical Sciences, Isfahan, Iran; 3 Department of Nursing and Midwifery, Islamic Azad University Isfahan (Khorasgan) Branch, Isfahan, Iran; 4 Department of Biostatistics and Epidemiology, School of Health, Isfahan University of Medical Sciences, Isfahan, Iran; 5 Department of Social & Behavioral Health, School of Public Health, & Department of Internal Medicine, University of Nevada, Las Vegas, USA

**Keywords:** L-carnitine, Sepsis, Inflammation, Oxidative stress, Mortality, Clinical trial

## Abstract

**Background:**

Sepsis, a life-threatening organ dysfunction caused by a host’s dysregulated response to infection with an inflammatory process, becomes a real challenge for the healthcare systems. L-carnitine (LC) has antioxidant and anti-inflammatory properties as in previous studies. Thus, we aimed to determine the effects of LC on inflammation, oxidative stress, and clinical parameters in critically ill septic patients.

**Methods:**

A randomized double-blinded controlled trial was conducted. A total of 60 patients were randomized to receive LC (3 g/day, *n* = 30) or placebo (*n* = 30) for 7 days. Inflammatory and oxidative stress parameters (C-reactive protein (CRP), erythrocyte sedimentation rate (ESR), superoxide dismutase (SOD), malondialdehyde (MDA), total antioxidant capacity (TAC), 28-day mortality rate, and some monitoring variables were evaluated.

**Results:**

There was no statistically significant difference between study arms in baseline characteristics and disease severity scores. CRP (*p* < 0.001) and ESR (*p*: 0.004) significantly reduced, and SOD (*p* < 0.001) and TAC (*p* < 0.001) significantly improved in the LC group after 7 days. Between-group analysis revealed a significant reduction in CRP (*p*: 0.001) and serum chloride (*p*: 0.032), an increase in serum albumin (*p*: 0.036) and platelet (*p*: 0.004) significantly, and an increase in SOD marginally (*p*: 0.073). The 28-day mortality rate was also lower in the LC group compared with placebo (7 persons vs. 15 persons) significantly (odds ratio: 0.233, *p*: 0.010).

**Conclusions:**

L-carnitine ameliorated inflammation, enhanced antioxidant defense, reduced mortality, and improved some clinical outcomes in critically ill patients with sepsis.

**Trial registration:**

IRCT20201129049534N1; May 2021.

## Introduction

Physiological, pathological, and biochemical abnormality in the body and organ dysfunction resulting from the dysregulated host immune response to infection is interpreted as sepsis syndrome [1]. Society of Critical Care Medicine (SCCM) clarifies the last definition of sepsis as “a life-threatening organ dysfunction caused by a host’s dysfunctional response to infection (sepsis-3)” [2]. As per the new guideline, the diagnosis of sepsis in infected or suspected patients in the intensive care unit (ICU) is based on the sequential organ failure assessment (SOFA) score. Sepsis is considered to have occurred when the total score is ≥ 2 [1]. In recent decades, sepsis has become a real challenge for the healthcare system and healthcare professionals. About 30 million patients suffer from sepsis yearly worldwide, and the incidence of sepsis among inpatients is 1–2% in charge of about half the inpatient fatality. In the United States, about 1 million sepsis cases are hospitalized yearly, and this tends to rise year by year [3]. Its mortality rate (in the USA) is predicted to be 12.5%. In the mentioned country, the cost of the burden of disease in 2013 was estimated to be over 20 billion dollars [1, 3].

Upper respiratory infection with *Pseudomonas sp.* is now the most frequent cause of sepsis [4]. Gram-negative and gram-positive bacteria, viral, fungal, and parasite agents also account for it. It’s noteworthy that in 30% of septic patients, the pathological infection cannot be detected. The diagnostic criteria of sepsis have changed over decades, but scientists always emphasize the presence of inflammation in the pathology of the disease [4]. Sepsis is commonly known as an inflammatory disease. Upon the entrance of a pathogen into the body, it releases certain molecules known as pathogen-associated molecular patterns (PAMPs). These PAMPs can then bind to pattern recognition receptors and trigger the activation of the innate immune system, protecting the invading pathogen. The induction of innate immunity causes the production of inflammatory cytokines and biomarkers that contribute to the systemic inflammatory response. Inflammatory cytokines, especially Interleukin-6 (IL-6), induce the release of acute phase proteins by the liver [5].

The C-reactive protein (CRP), a highly valuable laboratory biomarker, has been used for many years to determine inflammation. Under normal conditions, its concentration remains below 10 mg/L. However, when the immune system is stimulated, its levels can rapidly rise to as high as 10,000 mg/L within 1–2 days [6]. CRP can rise in infectious inflammatory diseases such as sepsis and non-infectious inflammatory conditions such as cardiovascular diseases or rheumatoid diseases. Thus, it is a non-specific biomarker for septic patients but is a sensitive marker to distinguish sepsis from non-septic causes of inflammation in the early onset of disease in the ICUs [7, 8]. Oxidative stress defined as unevenness between the production of reactive species reactive oxygen species (ROS), reactive nitrogen species (RNS) and antioxidants leads to the aggregation of the mentioned species in cells and injury to them. Sepsis can cause this imbalance and this imbalance also can worsen sepsis. Blood pressure of oxygen (PO_2_) in septic patients can be in the normal range, but it is believed that oxygen consumption may be reduced by septic peripheral cells. This condition is named ‘‘cytopathic hypoxia.’’ Cytopathic hypoxia during sepsis in a vicious cycle can lead to dysregulation in cellular energy production and the function of mitochondria along with worse outcomes for critically ill patients [9]. Although tens of studies have investigated putative treatments for sepsis, an effective remedy remains elusive [4]. Therefore, an effective agent is needed to ameliorate this ailment.

L-carnitine (LC) is a tertiary ammonium, and its well-known function is to facilitate the entrance of fatty acids to mitochondria for energy production. It can be synthesized endogenously or received from the diet [10]. LC supplementation not only has beneficial medical use in primary and secondary carnitine deficiency but is also prescribed broadly in cardiovascular diseases such as drug-induced myopathies, valproate toxicity, anorexia, chronic fatigue, male infertility, diphtheria, and drug-induced carnitine deficiencies [11]. Carnitine is synthesized from lysine and methionine with the help of vitamin C, vitamin B3, vitamin B6, and iron in hepatocytes, kidney, and brain cells. Despite being produced internally, stress conditions can lead to insufficient production, necessitating dietary sources or supplementation [12]. LC could increase the expression of superoxide dismutase-2 (SOD-2), reduce inflammation, and alleviate oxidative stress in animal models [13]. Recent evidence revealed that LC is a protectant agent of enzymes from oxidative damage through free radical scavenging and can enhance the levels of antioxidant enzyme activities [14]. As discussed, carnitine utilization is vital for the swing from carbohydrate to fat metabolism during the sepsis energy crisis. This is the basis for the rationale of LC supplementation as a therapeutic agent in sepsis [15].

Although recent studies revealed the benefits of LC supplementation in a variety of conditions even in critically ill patients, a lack of knowledge on the results of LC supplementation in sepsis persuaded us to design a novel trial. Therefore, this study aimed to investigate the effects of LC supplementation on inflammatory mediators, oxidative status, and monitoring parameters in septic ICU-admitted patients.

## Materials and methods

This study was approved by the Vice-Chancellor in Research Affairs -Medical University of Isfahan (Biomedical Research Ethics Committee) (approval code: IR.MUI.RESEARCH.REC.1400.037, March 2021). The Ethics Committee was responsible for monitoring the trial. Audits on accuracy were carried out twice during the trial.

Patients gave written informed consent and this trial was conducted by the Declaration of Helsinki principles [16]. This trial was also registered in the Iranian Registry of Clinical Trials (ID: IRCT20201129049534N1, May 2021, https://fa.irct.ir/trial/55874).

### Trial design

The full study protocol was previously published [17]. In summary, it was a parallel randomized, double-blinded, and clinical controlled trial and was conducted et al.-Zahra Hospital, Isfahan, Iran. Recruitment of participants was carried out between September 2021 and February 2023 in the general ICU.

### Participants

Eligibility criteria for participating in the study were: Septic ICU resident patients (diagnosed by sepsis-3 criteria), older than 18 years, having provided written informed consent, and being nourished enterally. Pregnant women, extremely low-weight persons (body mass index (BMI) < 18.5 kg/m^2^), patients who required frequent blood transfusions, septic shock patients, and those patients having any unwanted side effects after taking a supplement or placebo were excluded.

This trial was conducted et al.-Zahra Hospital, Isfahan, Iran.

For included participants, after hemodynamic resuscitation and stabilization, nutritional support with enteral tube feeding (25 kcal/kg of energy) was begun. Nutritional feeding was administered via bolus method (7 times in 24 h).

### Interventions

At first, the principal investigator (MK) explained the potential benefits of the current trial and obtained informed consent from their patients or their legal representatives. Then, they were randomized to receive a high dosage of LC (3 g/day, 1 gr t.i.d, in capsule form) or placebo which contained maltodextrin (3 g/day, 1 gr t.i.d, in capsule form) for 7 days.

Sealed and opaque envelopes were provided by the supplement manufacturer which determined A or B box content (LC or placebo).

Standard protocol treatment was implemented for all patients, our intervention added to those and did not need to alter the usual treatment strategy. Intervention and control capsules had similar shapes, sizes, colors, odors, and tastes. Capsules were packaged in pill boxes that were tagged A and B for blinding. Intervention and placebo capsules and blinded pillboxes were made by Karen Pharma and Food Supplement Company, Tehran, Iran.

### Outcomes

At baseline, disease severity status was evaluated using acute physiology and chronic health evaluation II (APACHE II), SOFA, quick SOFA (qSOFA), and nutrition risk in the critically ill (NUTRIC) score. Variations in inflammatory mediators were monitored by serum CRP and erythrocyte sedimentation rate (ESR) as principal outcomes at baseline and 7th day. The 28-mortality rate, oxidative status including total antioxidant capacity (TAC), malondialdehyde (MDA), SOD, and usual monitoring variables in the ICU monitoring were also appraised at baseline and endpoint of the study as secondary outcomes.

About 10 ml volume of fasting blood sample was drained before and after the study and then centrifuged, with the serum separated from the sediment, and preserved at a temperature of -80 °C. Laboratory personnel were unaware of intervention allocation.

The ELISA assay method has been used for assessing CRP, ESR, TAC, SOD activity, and MDA. Complete blood count (CBC) diff, blood urea nitrogen (BUN), creatinine (Cr), albumin (Alb), total bilirubin (Bill-T), direct bilirubin (Bil-D), prothrombin time (PT), and partial prothrombin time (PTT), were measured at the Clinical Chemistry Laboratory in Al-Zahra Hospital, according to a standardized protocol. Anthropometric variables also were evaluated by MK using a tape meter. Because of the limitations in the ICU weight and height were calculated using the Chumlea I formula [18] and an equation by Tarnowski, et al*.* [19] respectively. The 28-day mortality rate was collected and calculated by follow-up of patients after intervention by telephone contacting their families.

### Sample size

The sample size was calculated according to the results of a previous study [20] using the following equation and data:

$$n=\frac{2{({z}_{1-\alpha /2}+{z}_{1-\beta })}^{2}}{{\Delta }^{2}}+\frac{{z}_{1-\alpha /2}^{2}}{2}$$

$$\Delta =\frac{{\mu }_{1}-{\mu }_{2}}{\sigma }$$

mean of group 1 = 47.01.

mean of group 2 = 78.43.

σ = 42.

α = 0.05.

β = 0.2

Z_(1-α⁄2)_ = 1.96.

z_(1-β)_ = 0.84.

power = 80%

### Randomization

Intervention allocation was done by using a random number list and eligible patients were randomly allocated to trial groups in a ratio of 1:1.

### Allocation concealment mechanism

Sealed and opaque envelopes were provided by the supplement manufacturer which determined A or B box content (LC or placebo).

### Implementation

Intervention or control capsules were administered orally or with enteral nutrition (enteral tube feeding) three times a day at 9:00, 15:00, and 21:00.

### Blinding

The interventions were prescribed to participants using a double-blind method. It is noteworthy that patients or their legal guardians, all investigators, and data analysts were blinded.

### Statistical methods

All statistical analyses were performed based on the intention-to-treat (ITT) principle, in which missing values were imputed using multiple imputations. We used the expectation–maximization (EM) algorithm for missing data estimation. The data were entered into SPSS software version 21 and Stata software for analysis. The skewness test and Q–Q plots were applied to assess the normal distribution of variables. Quantitative and qualitative variables were reported as mean ± standard deviation (SD) and number (percentage), respectively. Baseline characteristics of the participants were compared between the groups, using the independent-sample t-test and Pearson’s chi-square test, where applicable. ANCOVA (Analysis of covariance) was used to detect any differences between the two groups at the end of the study and adjust for baseline values and the other confounders. The logarithmic transformation approach was applied to those variables with an abnormal distribution. For the 28-day mortality rate, we used a univariate and multivariable (adjusted for CRP and ESR values) logistic regression model. *p-*value < 0.05 was considered as significant.

## Results

### Participants characteristics

**Fig. 1:**
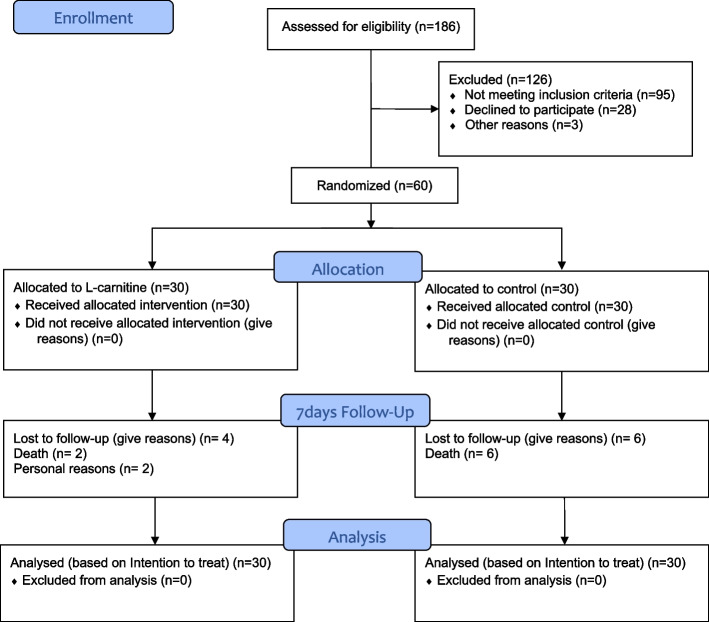
CONSORT study flow diagram. 30 out of 60 patients were allocated to the intervention group and 30 patients were allocated to the control group. Four persons in the intervention and 6 persons in the control group were lost to continue the trial but analysis was done on 60 persons based on the intention to treat (ITT) principals

In total, 186 patients were assessed for inclusion and exclusion criteria. Among them, 95 patients were excluded because they did not meet the inclusion criteria, 28 individuals refused to participate in the study, and 3 patients were excluded for individual reasons. Finally, 60 patients were randomly assigned to the intervention (3 g LC/day) or control (3 g placebo/day) group. Six patients in the control group and 2 patients in the LC group died during the study. Moreover, 2 participants in the LC group denied continuing the trial. Therefore, 26 participants in the LC group and 24 participants in the placebo group completed the trial but data analysis was based on ITT. All the remaining participants received their intervention (LC or placebo capsules) completely. Additionally, all patients were assessed by a blinded physician daily. There were no major adverse events in patients who received trial intervention. Only 2 participants, one in group A (LC) and one in group B (control) experienced some minor adverse effects such as stomachache and nausea. However, these symptoms were minor and acceptable for them and based on the physician's opinion, the intervention was continued for them. The CONSORT diagram of the current study is presented in Fig. 1.

The mean ± SD of age was 52.83 ± 17.71 years in LC and 53.33 ± 16.99 years in placebo groups (*p-*value*:* 0.912). In this study, 70% of the patients were men and 30% of them were women. The mean ± SD of the prognostic markers were 6.23 ± 2.37 in LC group and 7.13 ± 2.62 in the control group for SOFA (*p-*value*:* 0.169); 1.27 ± 0.45 in LC group and 1.50 ± 0.51 in the control group for qSOFA (*p-*value*:* 0.065); 18.50 ± 5.48 in LC group and 17.37 ± 4.71 in the control group for the APACHE II (*p-*value*:* 0.394); 3.93 ± 2.05 in LC group and 4.03 ± 1.83 in the control group (*p-*value*:* 0.843) for NUTIRC score. The primary site of infection analysis for recruited patients revealed that the majority of the participants had respiratory infections and were distributed equally to the intervention groups (Table 1**).** The baseline characteristics of the critically ill patients were comparable between the two groups **(**Table 1**)**.

**Table 1:** Baseline characteristics of intervention group (L-carnitine) and control group (placebo)

**Parameters**	**L-carnitine (*n* = 30), mean** ± **SD**	**Placebo (*n* = 30), mean** ± **SD**	***p-*value**^1^
**Age (year)**	52.83 ± 17.71	53.33 ± 16.99	0.912
***Sex***			
*** Male***	20 (66.7%)	22 (73.3%)	0.573
*** Female***	10 (33.3%)	8 (26.7%)	
**Height (cm)**	169.03 ± 4.95	169.79 ± 4.69	0.545
**Weight (kg)**	69.22 ± 17.46	67.41 ± 13.39	0.654
**BMI (kg/m**^**2**^**)**	24.16 ± 5.69	23.36 ± 4.32	0.545
**GCS**	6.53 ± 2.54	7.27 ± 2.54	0.269
**Heart rate**	98.03 ± 19.36	94.83 ± 23.07	0.563
**Respiratory rate**	19.83 ± 5.17	22.80 ± 15.09	0.313
**Calf circumference (cm)**	32.67 ± 4.67	32.47 ± 3.93	0.858
**MAC (cm)**	32.10 ± 4.99	31.17 ± 4.15	0.434
**Ulna (cm)**	26.87 ± 2.22	27.10 ± 2.41	0.698
**SOFA**	6.23 ± 2.37	7.13 ± 2.62	0.169
**qSOFA**	1.27 ± 0.45	1.50 ± 0.51	0.065
**APACHE II**	18.50 ± 5.48	17.37 ± 4.71	0.394
**Nutric score**	3.93 ± 2.05	4.03 ± 1.83	0.843
**The primary site of infection**			
** Respiratory**	15 (50.00%)	20 (66.70%)	0.190
** Urinary**	5 (16.70%)	3 (10.00%)	0.448
** Bloodstream**	3 (10.00%)	3 (10.00%)	0.999
** Abdominal**	3 (10.00%)	1 (3.30%)	0.301
** Other**	4 (13.30%)	3 (10.00%)	0.688

Continuous and categorical data are presented as Mean ± SD and frequency (percentage)

*BMI* Body mass index, *GCS* Glasgow coma scale, *MAC* Mean arm circumference, *SOFA* Sequential organ failure assessment, *qSOFA* Quick SOFA

^1^Resulting from Independent Samples t-test for continuous and Pearson Chi-Squared test for categorical variables

### The effects of LC on primary outcomes

In comparison to the baseline, CRP (54.43 ± 29.11 vs. 77.20 ± 28.23, *p-*value < 0.001) and ESR (72.37 ± 23.99 vs. 60.19 ± 25.37, *p-*value*:* 0.004) significantly reduced in the LC group after 7 days. Between-group analysis indicated that differences between the LC group and control group were significant for CRP (-22.77 ± 25.40 vs. 1.02 ± 21.10, *p-*value*:* 0.001) and were not significant for ESR (-12.18 ± 21.16 vs. 0.67 ± 22.43, *p-*value*:* 0.151), respectively (Table 2).

**Table 2:** Inflammation and oxidative stress variables in the intervention group (L-carnitine) and control group (placebo) at baseline and end of the trial (day 7)

**Variable**^a^		**Study Timeline**	**L-carnitine (*n* = 30), mean** ± **SD)**	**Placebo (*n* = 30), mean** ± **SD**	***p-*value**^2^	***p-*value**^3^	***p-*value**^4^
**Inflammation status**	***CRP (mg/dl)***	Baseline	77.20 ± 28.23	63.50 ± 36.60	0.110	0.001	0.001
		Endpoint	54.43 ± 29.11	64.51 ± 29.20			
		Mean difference ± SD	-22.77 ± 25.40	1.02 ± 21.10			
		*p-*value^1^	< 0.001	0.794			
	***ESR (mm/hr)***	Baseline	72.37 ± 23.99	53.47 ± 29.43	0.008	0.151	0.151
		Endpoint	60.19 ± 25.37	54.14 ± 32.71			
		Mean difference ± SD	-12.18 ± 21.16	0.67 ± 22.43			
		*p-*value	0.004	0.871			
**Oxidative stress status**	***TAC (nmol/ml)***	Baseline	4.23 ± 1.09	4.14 ± 0.96	0.679	0.821	0.835
		Endpoint	5.06 ± 1.40	5.04 ± 0.95			
		Mean difference ± SD	0.83 ± 1.95	0.90 ± 1.37			
		*p-*value	< 0.001	< 0.001			
	***MDA (nmol/ml)***	Baseline	32.21 ± 6.19	32.10 ± 7.33	0.829	0.814	0.879
		Endpoint	32.78 ± 6.55	32.39 ± 6.13			
		Mean difference ± SD	0.58 ± 4.05	0.29 ± 2.82			
		*p-*value	0.501	0.215			
	***SOD activity (U/ml)***	Baseline	18.02 ± 13.34	20.75 ± 15.59	0.402	0.071	0.050
		Endpoint	23.25 ± 11.96	19.17 ± 10.05			
		Mean difference ± SD	5.23 ± 11.97	-1.58 ± 17.32			
		*p-*value	< 0.001	< 0.001			

*CRP* C reactive protein, *ESR* Erythrocyte sedimentation rate, *TAC* Total antioxidants capacity, *SD* standard deviation

^1^Obtained from Paired sample t-test

^2^Obtained from Independent Sample t-test

^3^Obtained from analysis of covariance (ANCOVA) in the adjusted models (adjusted for baseline value)

^4^Obtained from analysis of covariance (ANCOVA) in the adjusted models (adjusted for baseline value and ESR)

^a^Values are Mean ± Standard deviation

The levels of TAC (5.06 ± 1.40 vs. 4.23 ± 1.09, *p-*value < 0.001) and SOD (23.25 ± 11.96 vs. 18.02 ± 13.34, *p-*value < 0.001) in the LC group were found to be significantly higher after the intervention, compared to their baseline levels. In comparison to the control group, the LC group showed a slight improvement in SOD levels (5.23 ± 11.97 vs. -1.58 ± 17.32, *p-*value*:* 0.071). However, there were no significant differences observed in other variables.

### The effects of LC on secondary outcomes

The results of the chi-square test revealed that the 28-day mortality rate was lower in the intervention group in comparison to the control group (7 persons (23.33%) vs. 15 persons (50.00%), *p-*value*:* 0.032). The results of multivariate logistic regression showed that intervention can reduce the risk of the 28-day mortality rate by 76% (odds ratio: 0.233, p-value: 0.010, 95% CI: 0.077 to 0.708) (Table 3).

**Table 3:** 28-day mortality rate analysis

28-day mortality rate	*p*-value	Odds ratio	95% CI
**Univariate test**^a^	0.012	0.184	0.049 to 0.688
**Multivariate test**^b^	0.010	0.233	0.077 to 0.708

^a^Crude model

^b^Adjusted for C-reactive protein (CRP) and erythrocyte sedimentation rate (ESR) values

Serum Alb (2.66 ± 0.50 vs. 2.55 ± 0.48, *p-*value*:* 0.025), Ca (7.93 ± 0.74 vs. 7.27 ± 0.73, *p-*value*:* 0.049), mean corpuscular volume (MCH) (27.75 ± 2.45 vs. 27.69 ± 2.55, *p-*value < 0.001), pH (7.47 ± 0.12 vs. 7.42 ± 0.08, *p-*value < 0.001), and PTT (32.98 ± 9.38 vs. 30.21 ± 5.08, *p-*value < 0.001) were significantly increased and PO_2_ (91.07 ± 29.08 vs. 110.95 ± 36.29, *p-*value*:* 0.005), body temperature (37.40 ± 0.48 vs. 37.77 ± 0.97, *p-*value*:* 0.012), systolic blood pressure (SBP) (117.05 ± 20.72 vs. 126.63 ± 21.87, *p-*value*:* 0.024), and diastolic blood pressure (DBP) (76.29 ± 7.93 vs. 85.30 ± 21.71, *p-*value*:* 0.012) were significantly reduced in LC group after 7 days of intervention. Moreover, MCHC (33.91 ± 2.24 vs. 33.11 ± 1.38, *p-*value*:* 0.081) and platelet (Plt) (276.26 ± 110.86 vs. 241.47 ± 116.47, *p-*value*:* 0.093) were marginally increased, and white blood cell (WBC) was marginally reduced (8.95 ± 3.13 vs. 9.96 ± 3.75, *p-*value*:* 0.059) in this group. Between-group analysis revealed that serum Alb (0.11 ± 0.26 vs. -0.06 ± 0.26, *p-*value*:* 0.036) and Plt (34.80 ± 109.82 vs. -39.13 ± 98.67, *p-*value*:* 0.004) were increased significantly after intervention in the LC group in comparison to the placebo group. Regarding the SBP, the value was reduced in both groups but the reduction in the control group was more (-14.99 ± 24.16 vs. -9.58 ± 21.98, *p-*value*:* 0.030). The mean levels of monitoring variables before and after intervention are shown in Table 4.

**Table 4:** Monitoring laboratory variables in the intervention group (L-carnitine) and control group (placebo) at baseline and end of the trial (day 7)

Variable^**a**^		Study Timeline	L-carnitine (*n* = 30), mean ± SD)	Placebo (*n* = 30), mean ± SD)	*p-*value^2^	*p-*value^3^	*p-*value^4^
**Hepatic status**	***ALT (U/L)***	Baseline	36.43 ± 20.13	42.20 ± 38.49	0.953	0.430	0.303
		Endpoint	40.44 ± 32.33	38.90 ± 34.79			
		Mean difference ± SD	4.01 ± 26.44	-3.30 ± 8.28			
		*p-*value	0.789	0.284			
	***AST (U/L)***	Baseline	42.20 ± 24.59	47.23 ± 38.63	0.879	0.587	0.672
		Endpoint	40.20 ± 21.98	44.50 ± 27.43			
		Mean difference ± SD	-2.00 ± 27.37	-2.74 ± 21.86			
		*p-*value	0.571	0.919			
	***ALP (U/L)***	Baseline	255.77 ± 168.94	265.70 ± 174.59	0.617	0.532	0.327
		Endpoint	263.09 ± 168.86	288.82 ± 193.23			
		Mean difference ± SD	7.32 ± 48.79	23.12 ± 49.96			
		*p-*value	0.645	0.114			
**Biochemistry tests**	***BUN (mg/dl)***	Baseline	22.37 ± 21.38	24.20 ± 18.90	0.337	0.971	0.965
		Endpoint	19.28 ± 12.00	19.57 ± 10.23			
		Mean difference ± SD	-3.09 ± 15.18	-4.63 ± 12.59			
		*p-*value	0.903	0.284			
	***Cr (mg/dl)***	Baseline	1.03 ± 0.49	1.43 ± 0.82	0.038	0.218	0.470
		Endpoint	1.08 ± 0.48	1.22 ± 0.60			
		Mean difference ± SD	0.05 ± 0.42	-0.21 ± 0.56			
		*p-*value	0.292	0.062			
	***Alb (g/dl)***	Baseline	2.55 ± 0.48	2.69 ± 0.65	0.368	0.036	0.027
		Endpoint	2.66 ± 0.50	2.63 ± 0.56			
		Mean difference ± SD	0.11 ± 0.26	-0.06 ± 0.26			
		*p-*value	0.025	0.343			
	***BS (mg/dl)***	Baseline	140.77 ± 46.64	120.47 ± 46.77	0.064	0.615	0.526
		Endpoint	147.04 ± 73.27	119.59 ± 38.99			
		Mean difference ± SD	6.27 ± 62.95	-0.88 ± 22.81			
		*p-*value	0.976	0.743			
	***Total Bilirubin (mg/dl)***	Baseline	1.06 ± 1.37	1.30 ± 1.21	0.062	0.462	0.532
		Endpoint	0.78 ± 0.25	1.02 ± 0.59			
		Mean difference ± SD	-0.29 ± 1.34	-0.28 ± 1.09			
		*p-*value	0.602	0.106			
	***Direct Bilirubin (mg/dl)***	Baseline	0.32 ± 0.17	0.57 ± 0.74	0.018	0.626	0.576
		Endpoint	0.28 ± 0.12	0.41 ± 0.40			
		Mean difference ± SD	-0.02 ± 0.07	-0.16 ± 0.39			
		*p-*value	0.719	0.072			
	***Na (mEq/L)***	Baseline	139.47 ± 4.30	138.20 ± 5.52	0.326	0.173	0.300
		Endpoint	139.60 ± 4.97	137.75 ± 3.54			
		Mean difference ± SD	0.13 ± 5.29	-0.45 ± 5.38			
		*p-*value	0.892	0.649			
	***K (mEq/L)***	Baseline	3.91 ± 0.42	4.05 ± 0.47	0.224	0.581	0.292
		Endpoint	4.01 ± 0.38)	4.11 ± 0.61)			
		Mean difference ± SD	0.10 ± 0.51	0.05 ± 0.73			
		*p-*value	0.290	0.688			
	***P (mg/dl)***	Baseline	3.04 ± 0.75	3.12 ± 1.01	0.717	0.249	0.212
		Endpoint	2.96 ± 0.67	2.86 ± 0.48			
		Mean difference ± SD	-0.83 ± 0.58	-0.26 ± 0.75			
		*p-*value	0.441	0.062			
	***Mg (mg/dl)***	Baseline	1.91 ± 0.35	1.93 ± 0.41	0.839	0.914	0.911
		Endpoint	1.89 ± 0.32	1.91 ± 0.43			
		Mean difference ± SD	-0.02 ± 0.30	-0.02 ± 0.50			
		*p-*value	0.722	0.812			
	***Ca (mg/dl)***	Baseline	7.27 ± 0.73	8.07 ± 0.81	0.091	0.360	0.686
		Endpoint	7.93 ± 0.74	8.06 ± 0.62			
		Mean difference ± SD	0.20 ± 0.53	-0.02 ± 0.45			
		*p-*value	0.049	0.856			
**CBC diff test**	***RBC (10***^***12***^***/L)***	Baseline	3.49 ± 0.55	3.70 ± 0.71	0.252	0.967	0.788
		Endpoint	3.56 ± 0.65	3.65 ± 0.54			
		Mean difference ± SD	0.07 ± 0.56	-0.06 ± 0.56			
		*p-*value	0.527	0.779			
	***WBC (10***^***9***^***/L)***	Baseline	9.96 ± 3.75	8.23 ± 3.06	0.059	0.290	0.154
		Endpoint	8.95 ± 3.13	8.82 ± 3.94			
		Mean difference ± SD	-1.01 ± 2.54	0.59 ± 3.91			
		*p-*value	0.059	0.366			
	***Lymphocyte (%)***	Baseline	13.65 ± 8.11)	15.32 ± 9.25	0.444	0.113	0.193
		Endpoint	13.46 ± 6.54	17.74 ± 10.81			
		Mean difference ± SD	-0.20 ± 7.22	2.42 ± 12.00			
		*p-*value	0.744	0.178			
	***Neutrophil (%)***	Baseline	73.13 ± 16.53	74.56 ± 11.51	0.871	0.167	0.116
		Endpoint	74.69 ± 10.97	70.80 ± 15.23			
		Mean difference ± SD	1.56 ± 10.67	-3.75 ± 16.40			
		*p-*value	0.727	0.207			
	***Hb (g/dl)***	Baseline	9.74 ± 1.53	10.31 ± 1.62	0.220	0.675	0.741
		Endpoint	9.76 ± 1.48	10.20 ± 2.00			
		Mean difference ± SD	0.01 ± 1.58	-0.12 ± 1.36			
		*p-*value	0.996	0.645			
	***Hct (%)***	Baseline	28.86 ± 3.86	31.38 ± 5.86	0.054	0.405	0.409
		Endpoint	29.05 ± 3.85	31.05 ± 4.55			
		Mean difference ± SD	0.19 ± 4.30	-0.34 ± 4.34			
		*p-*value	0.810	0.675			
	***MCV (fL)***	Baseline	83.12 ± 6.34	83.81 ± 10.43	0.295	0.316	0.435
		Endpoint	83.20 ± 6.41	85.41 ± 3.58			
		Mean difference ± SD	0.08 ± 3.56	1.60 ± 10.06			
		*p-*value	0.891	0.852			
	***MCH (pg)***	Baseline	27.69 ± 2.55	28.02 ± 2.30	0.589	0.174	0.421
		Endpoint	27.75 ± 2.45	28.44 ± 2.71			
		Mean difference ± SD	0.06 ± 1.15	0.42 ± 1.09			
		*p-*value	< 0.001	< 0.001			
	***MCHC (g/dl)***	Baseline	33.11 ± 1.38	32.47 ± 1.68	0.114	0.689	0.616
		Endpoint	33.91 ± 2.24	33.91 ± 3.03			
		Mean difference ± SD	0.80 ± 2.41	1.43 ± 2.99			
		*p-*value	0.081	0.014			
	***Plt (/ml)***	Baseline	241.47 ± 116.47	260.13 ± 127.15	0.556	0.004	0.042
		Endpoint	276.26 ± 110.86	220.30 ± 94.34			
		Mean difference ± SD	34.80 ± 109.82	-39.83 ± 98.67			
		*p-*value	0.093	0.035			
**ABG test**	***pH***	Baseline	7.42 ± 0.08	7.39 ± 0.09	0.207	0.999	1.000
		Endpoint	7.47 ± 0.12	7.44 ± 0.10			
		Mean difference ± SD	0.05 ± 0.13	0.05 ± 0.11			
		*p-*value	< 0.001	< 0.001			
	***PCO***_***2***_*** (mmHg)***	Baseline	41.70 ± 8.48	48.34 ± 15.76	0.110	0.633	0.809
		Endpoint	39.42 ± 9.66	43.18 ± 13.17			
		Mean difference ± SD	-2.28 ± 10.27	-5.16 ± 13.37			
		*p-*value	0.194	0.060			
	***PO***_***2***_*** (mmHg)***	Baseline	110.95 ± 36.29	94.27 ± 40.09	0.096	0.779	0.558
		Endpoint	91.07 ± 29.08	84.87 ± 25.43			
		Mean difference ± SD	-19.88 ± 36.18	-9.40 ± 40.08			
		*p-*value	0.005	0.209			
	***BE (mmol/L)***	Baseline	2.48 ± 6.30	3.63 ± 4.05	0.405	0.590	0.673
		Endpoint	2.27 ± 6.11	2.15 ± 5.10			
		Mean difference ± SD	-0.21 ± 6.32	-1.48 ± 4.76			
		*p-*value	0.856	0.099			
	***HCO***_***3***_*** (mmol/L)***	Baseline	26.39 ± 6.03	28.38 ± 4.84	0.165	0.610	0.731
		Endpoint	26.21 ± 6.81	27.77 ± 6.10			
		Mean difference ± SD	-0.18 ± 7.22	-0.61 ± 6.93			
		*p-*value	0.893	0.633			
**Coagulation status**	***PT (s)***	Baseline	13.71 ± 4.05	13.53 ± 3.66	0.976	0.443	0.767
		Endpoint	13.39 ± 2.87	13.49 ± 2.79			
		Mean difference ± SD	-0.32 ± 2.67	-0.04 ± 3.84			
		*p-*value	0.992	0.333			
	***PTT (s)***	Baseline	30.21 ± 5.08	33.29 ± 11.29	0.175	1.000	0.534
		Endpoint	32.98 ± 9.38	33.56 ± 10.53			
		Mean difference ± SD	2.78 ± 9.67	0.26 ± 10.53			
		*p-*value	< 0.001	< 0.001			
	***INR***	Baseline	1.29 ± 0.21	1.35 ± 0.37	0.709	0.180	0.346
		Endpoint	1.28 ± 0.18	1.35 ± 0.28			
		Mean difference ± SD	-0.01 ± 0.13	0.00 ± 0.38			
		*p-*value	0.841	0.145			
**Urinary status**	***SG***	Baseline	1018.83 ± 5.72	1020.10 ± 6.27	0.417	0.715	0.659
		Endpoint	1018.96 ± 5.20	1019.80 ± 5.30			
		Mean difference ± SD	0.127 ± 5.77	-0.31 ± 7.28			
		*p-*value	0.905	0.820			
	***Urinary pH***	Baseline	6.43 ± 1.07	6.23 ± 1.19	0.498	0.804	0.767
		Endpoint	6.43 ± 1.00	6.33 ± 0.98			
		Mean difference ± SD	0.000 ± 1.27	0.095 ± 1.34			
		*p-*value	0.998	0.701			
**Other monitoring variables**	***Temperature (°C)***	Baseline	37.77 ± 0.97	37.55 ± 0.72	0.321	0.151	0.269
		Endpoint	37.40 ± 0.48	37.45 ± 0.48			
		Mean difference ± SD	-0.37 ± 0.76	-0.10 ± 0.46			
		*p-*value	0.012	0.231			
	***SBP (mmHg)***	Baseline	126.63 ± 21.87	113.17 ± 17.94	0.012	0.030	0.007
		Endpoint	117.05 ± 20.72	98.17 ± 24.21			
		Mean difference ± SD	-9.58 ± 21.98	-14.99 ± 24.16			
		*p-*value	0.024	0.002			
	***DBP (mmHg)***	Baseline	85.30 ± 21.71	92.37 ± 27.76	0.277	0.393	0.027
		Endpoint	76.29 ± 7.93	75.53 ± 8.44			
		Mean difference ± SD	-9.02 ± 18.33	-16.84 ± 26.65			
		*p-*value	0.012	0.002			
	***PRL (ng/ml)***	Baseline	59.37 ± 32.87	48.05 ± 20.62	0.006	0.236	0.197
		Endpoint	55.61 ± 27.06	44.95 ± 17.89			
		Mean difference ± SD	-3.76 ± 31.76	-3.10 ± 21.77			
		*p-*value	0.521	0.442			

*ABG* arterial blood gas, *Alb* Albumin, *ALP* Alkaline phosphatase, *ALT* alanine aminotransferase, *AST* aspartate aminotransferase, *BE* Base Excess, *BS* Blood sugar, *BUN* Blood urea nitrogen, *Ca* Calcium, *CBC diff* Complete blood count with differential, *Cl* Chloride, *Cr* Creatinine, *DBP* Diastolic blood pressure, *Hb* Hemoglobin, *HCO3* bicarbonate, *Hct* Hematocrit, *INR* International normalized ration, *K* Potassium, *MCH* Mean corpuscular hemoglobin, *MCHC* Mean corpuscular hemoglobin concentration, *MCV* Mean corpuscular volume, *Mg* Magnesium; *Na* Sodium, *P* Phosphorus, *PCO2* Partial pressure of carbon dioxide, *Plt* Platelets, *PO2* partial pressure of oxygen, *PT* Prothrombin time, *PTT* Partial thromboplastin time, *RBC* Red blood cells, *SBP* Systolic blood pressure, *SG* Specific gravity, *WBC* White blood cells, *SD* standard deviation

^1^Obtained from Paired sample t-test

^2^Obtained from Independent Sample t-test

^3^Obtained from analysis of covariance (ANCOVA) in the adjusted models (adjusted for baseline value)

^4^Obtained from analysis of covariance (ANCOVA) in the adjusted models (adjusted for baseline value and ESR)

^a^Values are Mean ± Standard deviation

## Discussion

To the best of our knowledge, only a limited number of clinical trials have explored the efficacy of LC in the treatment of sepsis. Most of these trials have focused on intravenous administration of the supplement. However, this study stands out as the pioneer in investigating the benefits of oral supplementation. Based on the findings of the present study, high-dosage supplementation of LC has demonstrated effectiveness in reducing inflammation and the 28-day mortality rate associated with sepsis. These results highlight the potential benefits of LC as a therapeutic intervention for sepsis, warranting further investigation and consideration in clinical practice.

Despite the development in healthcare systems and the new generation of drugs, sepsis continues to claim victims and the etiology of the disease continues to be enigmatic. While microbial infection serves as the primary cause of sepsis, inflammation assumes a pivotal role in the advancement and prognosis of the disease [4]. Additionally, higher CRP level is related to increased fatality rates in the general population and in chronic diseases [21]. Hepatocytes produce a pattern recognition receptor, CRP, for transcriptional controlling of IL-6. Its plasma levels are determined by the rate of production and underlying inflammation severity. Therefore, it is a good inflammatory and infectious biomarker. A study by Ingels, et al*.* revealed that higher CRP levels are associated with a higher risk of infection and a lower chance of ICU discharge [22]. Over the years, numerous studies have been conducted to investigate the potential of anti-inflammatory agents in septic patients. However, the treatment outcomes from these studies have been less than satisfactory, and the results have remained inconclusive.

The first interventional study on LC was carried out on a rat’s sepsis model in 1989 which cleared mortality was reduced in the LC treatment group [10]. In an interventional animal trial by Kalhori, et al*.* on polycystic ovary syndrome-induced mice, LC in the dosage of 500 mg/kg for every second day demonstrated a significant reduction in IL-6, MDA, and tumor necrosis factor- α (TNF-α) in the intervention group versus control after 28 days [23]. According to a clinical trial conducted by Dastan et al*.*, administering 3 g of LC for a period of 4 days (2 days prior to surgery and 2 days after surgery) to patients undergoing coronary artery bypass graft surgery can significantly lower the incidence of arterial fibrillation and reduce serum CRP levels [24]. Original research by Derosa, et al*.*, prescribed LC supplements to 258 type-2 diabetes patients in 2 g dosage along with 360 mg orlistat per day in comparison to orlistat only (360 mg/day) for 1 year. TNF-α and high-sensitive C-reactive protein reduced significantly in the intervention group [25]. Another trial conducted in 36 hemodialysis participants for 3 months demonstrated that 1 g LC per day significantly increased plasma carnitine 1.5 folds (P < 0.001) and CRP significantly decreased compared to the baseline (P < 0.01) and in comparison to the control group (P < 0.05) [26]. A randomized clinical trial carried out on critically ill patients with septic shock cleared that 12 g LC in the form of bolus infusion in 12 h in comparison to a placebo can decrease the 28-mortality rate statistically significantly [27]. A randomized ICU-based clinical trial by Yahyapoor, et al*.* which was published recently, revealed that 3 g LC per day for 7 days in the liquid form via enteral tube feeding in 54 critically ill patients, improved serum Alb and decreased CRP and IL-6 biomarkers in the LC group in comparison to the placebo group which is in line with our results. The major limitation of the mentioned trial was the broadly heterogeneous patients that were included but we focus on septic patients [20, 28]. Thirteen clinical trials on the topic of LC supplementation effects on inflammatory biomarkers were reviewed by Haghighatdoost, et al*.* The meta-analysis results of the mentioned study revealed that LC supplementation reduces CRP, IL-6, and TNF-α significantly [29]. Another meta-analysis regarding inflammation and oxidative stress, and LC supplementation on 44 trials conducted by Fathizadeh, et al*.* indicated that in agreement with our results, the level of CRP decreased significantly, besides IL-6, TNF-α, and MDA decreased and also SOD increased meaningfully [21]. So, the results of the current study confirm previous meta-analysis findings that supplementation with a dosage of more than 2 gr/day is more efficient in the anti-inflammatory effects [21, 29].

As evidence revealed, LC can downregulate the nuclear factor kappa B (NF-κB) pathway and suppress induced nitric oxide synthase (iNOS) protein expression. iNOS protein expression is related to nitric oxide (NO) production and can lead to septic shock and atherosclerosis. Eventually, LC can reduce the circulatory level of NO and prevent septic shock and over-inflammation [30]. Moreover, LC can up-regulate peroxisome proliferator-activated receptor-γ (PPAR-γ), which is a key factor in the regulation of hepatic inflammation [29]. LC functions as an antioxidant and reduces inflammation by protecting membranes from lipid peroxidation like MDA, increasing radical scavenging properties, and boosting mitochondrial antioxidant defense [31]. LC can potentially prevent formation of ROS, regulate cell redox status,

and activate some molecular pathways which modulate cellular homeostasis, especially in critical conditions. LC reduces pro-oxidant ROS-generating enzymes xanthine-oxidase (XO) and nicotinamide-adenine-dinucleotidephosphate-oxidase (NADPH-oxidase), and chelates metal ions (Fe2 + , Cu +), or acts as a buffer for excessive acetyl groups, that leads to a lower mitochondrial superoxide production [32]. LC acts as a mitochondrial antioxidant by preserving mitochondrial electron-transport-chain integrity [33]. Moreover, LC can potentially up-regulate SOD, glutathione-peroxidase (GSH-Px), catalase (CAT), and glutathione reductase (GR) through the pathway of regulating some transcriptional factors such as nuclear-factor-erythroid-2-related-factor-2 (Nrf2), PPAR-γ, PPAR-α, and NF-κB. The anti-oxidative activity of LC is believed to be primarily achieved by up-regulating the expression of heat-shock proteins, thioredoxin, and sirtuins. At the same time, LC is thought to down-regulate the expression of oxidative-stress-related genes such as Bax, Bcl-2, and caspase-3 [32]. As mentioned, LC is a key factor of the tricarboxylic acid cycle which enters fatty acids into the mitochondria for energy production. Therefore, oxygen concentration declined and ROS formation was also decreased [14].

Our results confirm that 3 gr LC/day for 1-week improved Alb level in septic patients. These findings are in agreement with the results of a clinical trial by Duranay, et al*.* conducted on 42 hemodialysis patients. The participants were supplemented with an infusion form of LC with a dosage of 20 mg/kg after each hemodialysis round (three times per week). After 6 months Alb and total protein significantly increased in the LC group but did not change in the control group [34]. A non-randomized clinical trial conducted on hemodialysis patients revealed that 15 mg/kg LC 3 times per week (after each hemodialysis session) for 6 months can increase Alb, total protein, glutathione (GSH), GSH peroxidase, and decrease MDA, but cannot change SOD and serum antioxidant capacity significantly [35]. In the current trial, increased serum Alb levels may be due to reduced inflammation, as Alb is a negative acute-phase protein. In other words, decreased inflammation leads to improved albumin levels [36].

Septic shock is sepsis with persistent hypotension (mean arterial blood pressure < 60 mmHg) and hypoperfusion where its mortality rate from sepsis is further high at 40–60%. In patients with hypotension, vasoactive agents are used to maintain arterial pressure. Lower blood pressure in these critically ill patients is along with adverse prognosis [37]. In a randomized clinical trial involving 115 participants with septic shock, LC supplementation was found to lead to notable improvements in hemodynamic parameters. Specifically, the study observed favorable changes in right atrial pressure and mean arterial blood pressure [38]. Contrary to the evidence, in the current study blood pressure of the participants was reduced but the reduction in the LC group was lower than in the control group. We hypothesized that a high dosage of LC could regulate blood pressure for its effects on improvement in cell oxygenation and energy metabolism.

This trial is the first in oral divided supplementation with a capsule form of LC in septic patients, otherwise recent studies were conducted in other patients with another form of LC. Other positive points of the current study include almost the full list of monitoring data. Bias was curtailed by allocation concealment, double blinding of participants, investigators, and laboratory staff, and an ITT analysis. Notwithstanding the novelty of the current study, it is essential to acknowledge several limitations. Firstly, the study was constrained by a relatively small number of participants, which could potentially impact the generalizability of the findings and its results should be interpreted with caution. Additionally, due to budgetary constraints, certain crucial assessments such as plasma carnitine levels and a wider array of inflammatory and cytokine biomarkers, and even serum lactate could not be carried out, which could have provided valuable insights. The disease severity scores could be assessed at the endpoint of the study. Furthermore, accurate measurements of weight and height were unfeasible due to limitations within our ICU unit. These limitations signify potential areas for improvement in future research endeavors aiming to build upon the insights gained from this study.

## Conclusion

The findings of the current randomized parallel controlled trial provide compelling evidence that high-dosage LC supplementation (3 gr/day) in ICU septic patients holds significant potential. Supplementation demonstrated the ability to ameliorate inflammation and boost anti-oxidative status, leading to improvements in certain clinical parameters. Most notably, the study observed a reduction in mortality after just 7 days of treatment. These encouraging results highlight the potential benefits of LC supplementation as a therapeutic intervention in managing sepsis and offer promising prospects for enhancing patient outcomes in critical care settings.

## Data Availability

No datasets were generated or analysed during the current study.

## Abbreviations

Alb: Albumin
ANCOVA: Analysis of covariance
APACHE II: Acute physiology and chronic health evaluation II
Bil-D: Direct bilirubin
Bill-T: Total bilirubin
BMI: Body mass index
BUN: Blood urea nitrogen
CAT: Catalase
CBC: Complete blood count
Cl: Chloride
Cr: Creatinine
CRP: C-reactive protein
DBP: Diastolic blood pressure
ESR: Erythrocyte sedimentation rate
GR: Glutathione reductase
GSH: Glutathione
GSH-Px: Glutathione-peroxidase
ICU: Intensive care unit
IL-6: Interleukin-6
iNOS: Induced nitric oxide synthase
ITT: Intention-to-treat
LC: L-carnitine
MCH: Mean corpuscular volume
MCHC: Mean corpuscular volume concentration
MDA: Malondialdehyde
NADPH-oxidase: Nicotinamide-adenine-dinucleotide phosphate-oxidase
Nrf2: Nuclear-factor-erythroid-2-related-factor-2
NF-kB: Nuclear factor kappa B
NO: Nitric oxide
NUTRIC: Nutrition risk in the critically ill
PAMPs: Pathogen-associated molecular patterns
Plt: Platelet
PO_2_: Blood pressure of oxygen
PPAR: Peroxisome-proliferator activated-receptor
PT: Prothrombin time
PTT: Partial prothrombin time
qSOFA: Quick SOFA
RNS: Reactive nitrogen species
ROS: Reactive oxygen species
SBP: Systolic blood pressure
SCCM: Society of critical care medicine
SD: Standard deviation
SOD: Superoxide dismutase
SOFA: Sequential organ failure assessment
TAC: Total antioxidant capacity
TNF-α: Tumor necrosis factor- α
WBC: White blood cell
XO: Xanthine-oxidase
